# Mild Subarachnoid Hemorrhage (SAH) With Low Hounsfield Unit Value (HUV) or Hijdra Scores Still Carries Outcome Risk: A Subgroup Analysis

**DOI:** 10.7759/cureus.99623

**Published:** 2025-12-19

**Authors:** Yushin Takemoto

**Affiliations:** 1 Neurosurgery, Japanese Red Cross Kumamoto Hospital, Kumamoto, JPN; 2 Neurosurgery, Tokuda Neurosurgical Hospital, Kanoya, JPN; 3 Neurosurgery, Kumamoto University Hospital, Kumamoto, JPN

**Keywords:** aneurysmal subarachnoid hemorrhage, delayed cerebral ischemia (dci), hounsfield unit value, intraventricular hemorrhage (ivh), symptomatic cerebral vasospasm

## Abstract

The prognostic value of the initial Hounsfield unit value (IT-HUV) in aneurysmal subarachnoid hemorrhage (SAH) has been demonstrated in previous work, but it remains uncertain whether patients with mild clinical presentation and low clot burden can truly be considered low risk. To address this question, we analyzed two subgroups of WFNS 1-2 patients: (1) limited hemorrhage volume (Hijdra 0-19; n = 46) and (2) low hemorrhage density (IT-HUV <46; n = 51). Despite their favorable initial appearance, both subgroups exhibited substantial rates of poor outcome, occurring in 13/46 (28.3%) and 15/51 (29.4%), respectively, while delayed cerebral ischemia (DCI) and symptomatic vasospasm (SVS) also occurred with nonnegligible frequency. Within these restricted cohorts, IT-HUV no longer demonstrated meaningful discriminatory ability, whereas age consistently remained the strongest predictor of outcome. Intraventricular hemorrhage contributed modest but clinically relevant additional risk information. These findings indicate that mild SAH accompanied by low-volume or low-density hemorrhage does not reliably correspond to a benign course. Elderly patients, particularly those with intraventricular extension, may still be vulnerable to complications and therefore warrant careful clinical monitoring rather than exclusion from vasospasm prophylaxis based solely on initial mild features.

## Editorial

Takemoto et al. previously reported that the initial Hounsfield unit value (IT-HUV) measured in the interpeduncular cistern is associated with symptomatic vasospasm (SVS), delayed cerebral ischemia (DCI), and functional outcome following aneurysmal subarachnoid hemorrhage (SAH) [1]. Building upon these findings, we performed additional subgroup analyses focusing on patients with both mild clinical grade (World Federation of Neurological Surgeons (WFNS) 1-2), as first proposed by Drake in 1988 [2], and limited clot burden (Hijdra 0-19), as defined by the Hijdra scale [3].

The choice of the 0-19 cutoff for the Hijdra sum score was based on two factors: (1) in our original cohort, the median Hijdra score was 19, making this threshold consistent with prior data; and (2) scores ≤19 are widely interpreted as representing a limited clot burden in clinical practice, allowing clinically meaningful stratification of mild SAH patients. Despite their seemingly favorable initial appearance, this subgroup demonstrated considerable variability in clinical outcomes.

Among the 46 patients meeting these criteria, poor outcome (modified Rankin scale (mRS) >2) occurred in 13/46 (28.3%), while DCI and SVS were observed in 3/46 (6.5%) and 2/46 (4.3%), respectively. Logistic regression revealed that age was the only independent predictor of poor outcome (OR: 1.10 per year; 95% CI: 1.02-1.18). Although intraventricular hemorrhage (IVH) showed a strong trend toward worse prognosis, IT-HUV did not predict DCI or SVS within this subgroup. The key characteristics of this subgroup are summarized in Table 1.

**Table 1:** Baseline characteristics of the mild/low-clot subgroup SAH: subarachnoid hemorrhage; WFNS: World Federation of Neurological Surgeons; IT-HUV: initial Hounsfield unit value; IVH: intraventricular hemorrhage; DCI: delayed cerebral ischemia; SVS: symptomatic vasospasm; mRS: modified Rankin scale

Variable	Value
Number of patients	46
Age (mean ± SD)	62.8 ± 13.9
IVH, n (%)	18 (39.1%)
IT-HUV, mean ± SD	28.6 ± 12.1
DCI, n (%)	3 (6.5%)
SVS, n (%)	2 (4.3%)
Poor outcome (mRS > 2), n (%)	13 (28.3%)

**Figure 1:**
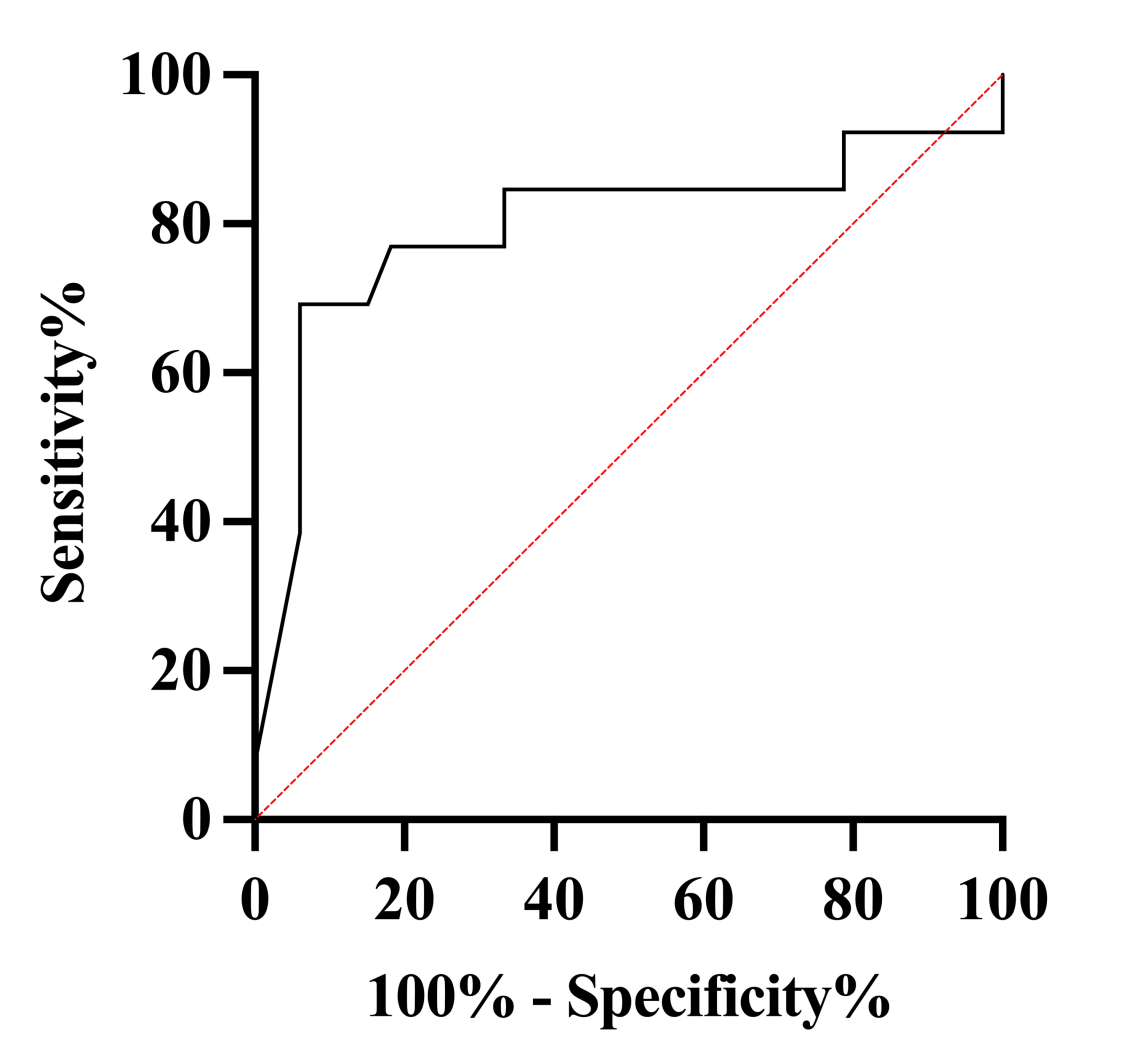
ROC curve: age predicting poor outcome ROC: receiver operating characteristic ROC curve evaluating age as a predictor of poor functional outcome (AUC = 0.80; 95% CI: 0.62-0.97)

Receiver operating characteristic (ROC) analyses further supported these findings: age demonstrated good discriminative ability (AUC: 0.80; 95% CI: 0.62-0.97). As shown in Figure 1, age provided substantial discriminatory power.

**Figure 2:**
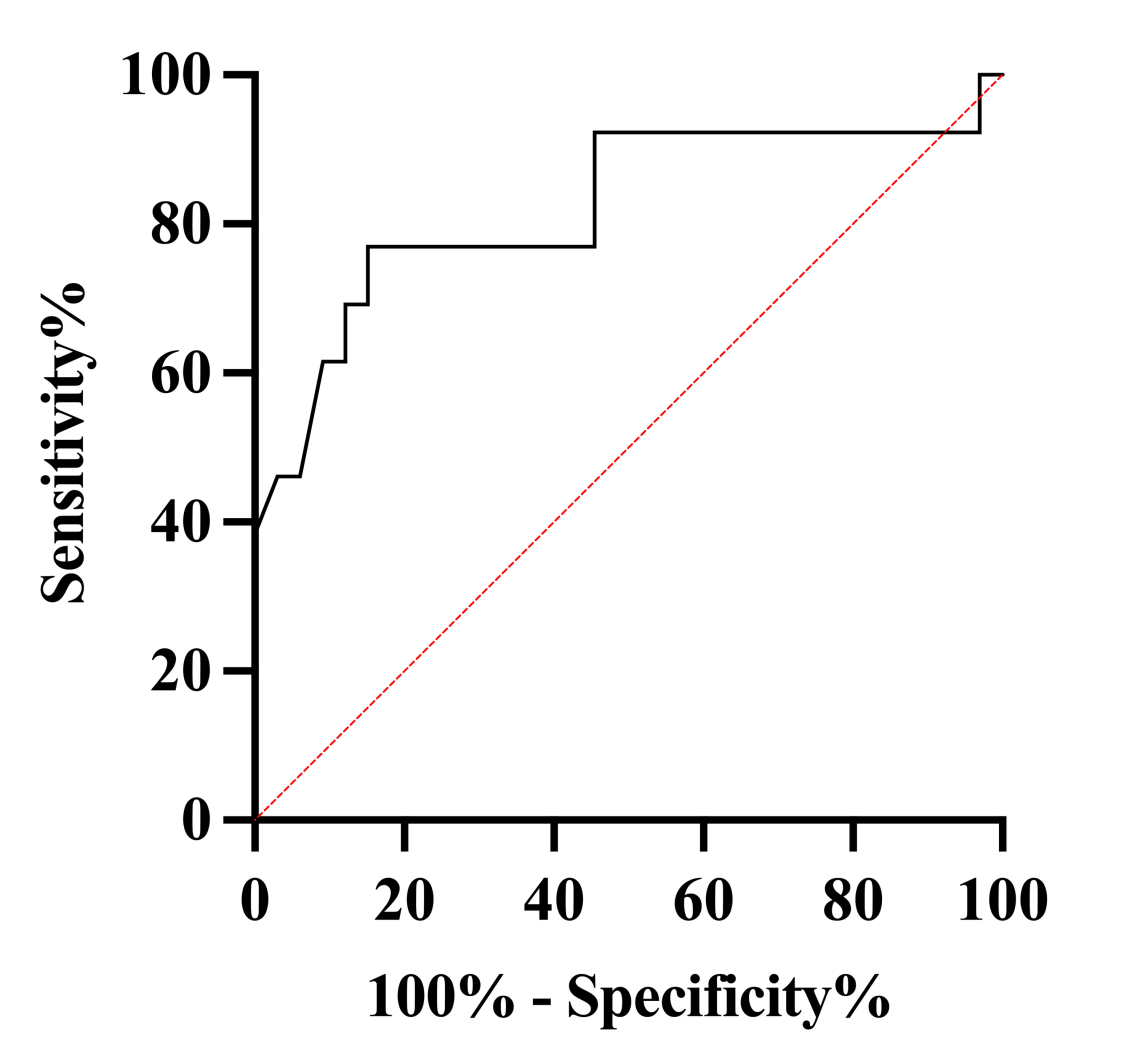
ROC curve: multivariate model (age + IVH) ROC: receiver operating characteristic; IVH: intraventricular hemorrhage ROC curve for simplified multivariate score (AUC = 0.82; 95% CI: 0.66-0.98)

A multivariate model incorporating age and IVH achieved an AUC of 0.82 (95% CI: 0.66-0.98; Figure 2). As this is an editorial, additional analyses such as calibration metrics or decision-curve analysis were beyond the intended scope.

These results indicate that even patients with mild neurological grade and low-volume hemorrhage may still experience clinically significant complications. To further assess whether hemorrhage density itself could define a low-risk subgroup, we additionally examined patients with WFNS 1-2 and IT-HUV < 46, the previously reported threshold associated with reduced risk [1]. Even within this ostensibly low-density subgroup (n = 51), poor outcome occurred in 15/51 (29.4%), with DCI and SVS observed in 4/51 (7.8%) and 3/51 (5.9%), respectively. 

Consistent with the findings in the Hijdra-based subgroup, IT-HUV again demonstrated no meaningful discriminatory capacity (AUC = 0.50), whereas age retained strong prognostic value (AUC = 0.82; 95% CI: 0.64-0.97). A multivariate model including age and IVH produced only marginal improvement (AUC = 0.83; 95% CI: 0.66-0.97). Thus, clot density alone does not reliably identify patients with truly benign trajectories. Taken together, these results indicate that mild SAH accompanied by low Hijdra scores or low IT-HUV values does not necessarily correspond to a benign clinical course, particularly among elderly patients or those with intraventricular extension.

Although patients with WFNS grade 1-2 SAH are commonly assumed to be at low risk, our analyses demonstrate that mild SAH represents a heterogeneous population in whom clinically significant complications may still occur. Age emerged as the strongest and most consistent predictor across analyses. This aligns with prior work, including Takemoto et al. [4], showing that age remains an independent predictor of poor functional outcome even after adjusting for hemorrhage burden.

The presence of IVH also warrants attention. Although not statistically significant in multivariable regression, IVH improved discriminative performance and has been associated with impaired CSF circulation, acute hydrocephalus, and worse functional outcomes in prior work by Zanaty et al. [5]. The trend seen in our cohort likely reflects these well-established pathophysiological mechanisms. 

In contrast, IT-HUV did not retain meaningful predictive value within restricted mild cohorts. This may reflect a threshold effect: when hemorrhage volume and density are already low, Hounsfield-based metrics exhibit limited variability and may not accurately represent the biological processes underlying delayed ischemia or poor outcomes.

In keeping with reviewer recommendations, all corresponding p-values and 95% confidence intervals for AUC values have been added throughout the manuscript to enhance statistical transparency and interpretability. Additionally, although WFNS, Hijdra, and mRS are open-access instruments, all have been fully cited according to journal requirements.

These subgroup analyses were exploratory in nature, and the relatively small sample sizes, multiple comparisons, and limited statistical power should be considered when interpreting the findings. This limitation does not detract from the clinical relevance of the results but instead reinforces the need for cautious interpretation and highlights opportunities for further investigation.

Altogether, our findings caution against assuming that mild SAH signifies a benign disease course. Elderly patients and those with IVH may still be vulnerable to deterioration, DCI, or functional decline and should not be excluded from careful monitoring or vasospasm prophylaxis solely based on favorable initial imaging. These results support a more nuanced risk-assessment framework and underscore the need for larger multicenter studies to validate whether age and IVH should be incorporated into future prognostic tools.
